# Case Report: Off-Label Liraglutide Use in Children With Wolfram Syndrome Type 1: Extensive Characterization of Four Patients

**DOI:** 10.3389/fped.2021.755365

**Published:** 2021-12-14

**Authors:** Giulio Frontino, Tara Raouf, Daniele Canarutto, Eva Tirelli, Raffaella Di Tonno, Andrea Rigamonti, Maria Lucia Cascavilla, Cristina Baldoli, Roberta Scotti, Letizia Leocani, Su-Chun Huang, Franco Meschi, Graziano Barera, Vania Broccoli, Greta Rossi, Silvia Torchio, Raniero Chimienti, Riccardo Bonfanti, Lorenzo Piemonti

**Affiliations:** ^1^Department of Pediatrics, IRCCS San Raffaele Hospital, Milan, Italy; ^2^Diabetes Research Institute, IRCCS San Raffaele Hospital, Milan, Italy; ^3^Vita-Salute San Raffaele University, Milan, Italy; ^4^San Raffaele Telethon Institute for Gene Therapy, IRCCS San Raffaele Hospital, Milan, Italy; ^5^Pediatric Immunohematology Unit and BMT Program, IRCCS San Raffaele Hospital, Milan, Italy; ^6^Department of Ophthalmology, IRCCS San Raffaele Hospital, Milan, Italy; ^7^Neuroradiology Unit, IRCCS San Raffaele Hospital, Milan, Italy; ^8^Experimental Neurophysiology Unit, Institute of Experimental Neurology, IRCCS San Raffaele Hospital, Milan, Italy; ^9^Institute of Neuroscience, National Research Council, IRCCS San Raffaele Hospital, Milan, Italy; ^10^Stem Cells and Neurogenesis Unit, IRCCS San Raffaele Hospital, Milan, Italy

**Keywords:** Wolfram syndrome, Wolfram syndrome 1 (WFS1), monogenic diabetes, GLP1 receptor agonists, liraglutide, neurodegeneration

## Abstract

**Aims:** Wolfram syndrome type 1 is a rare recessive monogenic form of insulin-dependent diabetes mellitus with progressive neurodegeneration, poor prognosis, and no cure. Based on preclinical evidence we hypothesized that liraglutide, a glucagon-like peptide-1 receptor agonist, may be repurposed for the off-label treatment of Wolfram Syndrome type 1. We initiated an off-label treatment to investigate the safety, tolerability, and efficacy of liraglutide in pediatric patients with Wolfram Syndrome type 1.

**Methods:** Pediatric patients with genetically confirmed Wolfram Syndrome type 1 were offered off-label treatment approved by The Regional Network Coordination Center for Rare Diseases, Pharmacological Research IRCCS Mario Negri, and the internal ethics committee. Four patients were enrolled; none refused nor were excluded or lost during follow-up. Liraglutide was administered as a daily subcutaneous injection. Starting dose was 0.3 mg/day. The dose was progressively increased as tolerated, up to the maximum dose of 1.8 mg/day. The primary outcome was evaluating the safety, tolerability, and efficacy of liraglutide in Wolfram Syndrome type 1 patients. Secondary endpoints were stabilization or improvement of C-peptide secretion as assessed by the mixed meal tolerance test. Exploratory endpoints were stabilization of neurological and neuro-ophthalmological degeneration, assessed by optical coherence tomography, electroretinogram, visual evoked potentials, and magnetic resonance imaging.

**Results:** Four patients aged between 10 and 14 years at baseline were treated with liraglutide for 8–27 months. Liraglutide was well-tolerated: all patients reached and maintained the maximum dose, and none withdrew from the study. Only minor transient gastrointestinal symptoms were reported. No alterations in pancreatic enzymes, calcitonin, or thyroid hormones were observed. At the latest follow-up, the C-peptide area under the curve ranged from 81 to 171% of baseline. Time in range improved in two patients. Neuro-ophthalmological and neurophysiological disease parameters remained stable at the latest follow-up.

**Conclusions:** We report preliminary data on the safety, tolerability, and efficacy of liraglutide in four pediatric patients with Wolfram Syndrome type 1. The apparent benefits both in terms of residual C-peptide secretion and neuro-ophthalmological disease progression warrant further studies on the repurposing of glucagon-like peptide-1 receptor agonists as disease-modifying agents for Wolfram Syndrome type 1.

## Introduction

Wolfram Syndrome type 1 (WS) is a rare autosomal recessive disease (OMIM: 222300) characterized by a variable combination of juvenile-onset of insulin-dependent diabetes mellitus (IDDM), diabetes insipidus, optic nerve atrophy, hearing loss, and progressive neurodegeneration (1, 2). Median survival is 39 years (range 25–49), and death occurs primarily due to progressive brain-stem atrophy-induced respiratory failure (3–5). The gene responsible for WS is *WFS1* which encodes for wolframin, a transmembrane glycoprotein involved in the regulation of the unfolded protein response (6). Dysfunctional wolframin results in endoplasmic reticulum (ER) stress, perturbed calcium homeostasis which, in turn, alters mitochondrial dynamics (7, 8). Apoptotic neuronal loss and beta cell death reflect the critical role of wolframin particularly in these cell types (9–11). WS is an orphan disease. While diabetes mellitus can be adequately managed, there is no effective treatment that slows the progression of neurodegeneration in WS. Glucagon-like peptide-1 receptor agonists (GLP-1RA) are commonly used for the treatment of type 2 diabetes with pleiotropic effects, ranging from improvement in glucose-dependent insulin secretion and beta cell function, delayed gastric emptying, and appetite reduction leading to loss of weight (12). Furthermore, recent studies have shown the neuroprotective effects of GLP-1RA in other neurodegenerative diseases such as Alzheimer's and Parkinson's disease (13, 14). Preclinical studies have shown the potential benefits of GLP-1RA in mitigating ER stress, which is thought to trigger the pathogenic cascade in WS (15, 16). Of note, liraglutide is known to cross the blood-brain barrier (17).

Liraglutide is the only GLP-1RA approved by the Food and Drug Administration and the European Medical Agency for children older than 10 affected by type 2 diabetes since 2019 (18, 19). Based on the favorable safety profile of the drug, both in type 2 diabetes (19, 20) and in a single adult WS patient (21), and preclinical evidence of the efficacy of GLP-1RA in WS murine models (22, 23), we hypothesized that liraglutide may improve the glycemic control of patients with WS, hamper the progression of neurodegeneration, and promote beta cell survival, assessed by C-peptide secretion. Therefore, we started an off-label treatment protocol with liraglutide for pediatric patients with WS. We report preliminary evidence of the safety, tolerability, and efficacy of liraglutide in four pediatric WS patients along with variations in glucometabolic, neuro-ophthalmic, neuroradiological and neurophysiological parameters.

## Diagnostic Assessment

### Patient Population

Patients older than 10 years with clinically and genetically confirmed WS presenting with IDDM and no contraindications for liraglutide use were eligible to start off-label treatment and follow-up at IRCCS San Raffaele Hospital in Milan, Italy. The off-label treatment protocol was approved by The Regional Network Coordination Center for Rare Diseases, Pharmacological Research IRCCS Mario Negri and by the internal ethics committee. Parenteral written informed consent was obtained, and patients provided verbal informed consent. Patients enrolled in the study were given off-label liraglutide in adjunct to the ongoing insulin therapy and other concomitant medications. Patients continued with regular scheduled pediatric diabetological visits. Dietitian and psychological consultations were completed as necessary. Patients were instructed to administer liraglutide subcutaneously via a pen injector device (Victoza) at dinnertime, at a starting dose of 0.6 mg/day (in two patients, liraglutide was started at 0.3 mg/day via U-100 subcutaneous syringe due to parental concern) and increased progressively up to the maximum deliverable dose of 1.8 mg/day. In case of moderate or severe adverse gastrointestinal symptoms upon dose escalation, patients were instructed to suspend treatment and contact our center before returning to the highest tolerated dose for another week and attempting to increase the dose again. Patients and caregivers were instructed to titrate insulin doses accordingly and briefed on the prevention and management of both mild and severe hypoglycemia. Follow-up of disease progression was based on the “Management of Wolfram Syndrome A Clinical Guideline Wolfram Syndrome Guideline Development Group” by the EURO-WABB Project (24). Follow-up visits were planned every 6 months, but schedules were readjusted as a consequence of the COVID-19 pandemic. Height, weight, and BMI were plotted on national reference growth curves (25). Six-points C-peptide area under the curve (C-AUC) was calculated with a 120-min mixed meal tolerance test (MMTT) with Nestle BOOST solution at 6 mL/kg, (Nestle HeathScience BOOST). Glucose time in range (TIR) defined as glucose values 70–180 mg/dL, time below range (TBR) defined as <70 mg/dL and time above range (TAR) defined as >180 mg/dL were evaluated in 28-day intervals by continuous glucose monitoring (CGM) (26). CGM monitoring was collected via Tidepool (Tidepool version 145.1) and Diasend (Diasend by Glooko software version R9b9).

### Neuro-Ophthalmology Assessment

All subjects underwent a complete neuro-ophthalmologic examination at baseline and in every follow-up visit. Optical Coherence Tomography (OCT) (CIRRUS OCT, software version 4.0.1; Carl Zeiss Meditec, Inc, Dublin, CA) was performed to measure the retinal nerve fiber layer (RNFL) and ganglion cell complex (GCC) after pharmacological mydriasis and OCT lenses were calibrated on patients' refractive error. For each eye, the mean RNFL thickness (360° measure), temporal quadrant thickness (316–45° unit circle), superior quadrant thickness (46–135°), nasal quadrant thickness (136–22°), and inferior quadrant thickness (226–315°) were automatically calculated by the endowed OCT software. Extended neuro-ophthalmological evaluations also included best-corrected visual acuity measurement (BVCA), pattern electroretinography (PERG), Ishihara test, Humphrey 30-2 visual field examination, slitlamp biomicroscopy, intraocular pressure measurement, and indirect ophthalmoscopy.

### Neuroradiology Assessment

All magnetic resonance imaging (MRI) acquisitions were conducted on a 1.5 Tesla Philips Intera scanner (Philips Medical System, Eindhoven, The Netherlands). The scanning protocol included a standardized pediatric brain MRI protocol consisting of axial, coronal, and sagittal T1 and T2-weighted sequences, volumetric T1- weighted, fluid-attenuated inversion recovery, and diffusion-weighted imaging sequences. An orbit protocol consisting of T2, T2 fat sat, and T1 sequences on the coronal and axial plane were also obtained. The evaluation consisted of a brain morphological assessment, particularly regarding pontine dimension, signal intensity, and optic radiations alteration. Optic nerves, optic chiasm, and optic tracts dimension and signal intensity were also evaluated. The hypothalamic-pituitary region was included in the evaluation.

### Neurophysiology Assessment

Visual pathway function was monitored with multi-focal visual evoked potentials (mfVEP), recorded using Accumap (ObjectiVision Pty. Ltd., Sydney, New South Wales, Australia) (27). Dartboard pattern-reversal stimuli were delivered over 56 concentric sectors of the visual field for each eye with a pseudorandom sequence. The recordings were performed monocularly and refractive corrections were used when needed. The signals were recorded with four surface gold cup electrodes placed around the inion, to create 2 bipolar traces (vertical and horizontal) for each sector. Bipolar traces corresponding to the cortical response for each sector were analyzed with in-house scripts developed with Matlab (Mathwork, Natick, MA). For each eye, mfVEPs for all 56 sectors of the visual field were averaged into one trace and the amplitude was quantified as the signal-to-noise ratio (SNR) of the averaged trace (28). Low contrast visual acuity (LCVA) at 2.5% of contrast was measured monocularly with a Sloan chart, recording the number of correct letters identified for each eye.

### Statistical Analysis

No statistical analysis was performed. All graphs were created using Prism 9 (GraphPad software 2021).

## Results

Four consecutive patients affected by WS were offered to start off-label treatment. Signed informed consent was collected, and treatment was started after exclusion of any possible contraindication to liraglutide treatment. All patients were born from non-consanguineous parents, who had been found to be carriers of the disease and all families were offered genetic counseling. Patients' characteristics are summarized in Table 1. In all cases, IDDM presented at onset without ketoacidosis; glycated hemoglobin (HbA1c) at diagnosis ranged from 9.4 to 16.7% (79–159 mmol/mol). All patients tested negative for type 1 diabetes mellitus related autoantibodies (Glutamic acid decarboxylase autoantibodies, Insulinoma-associated-2 autoantibodies, Zinc Transporter 8, and Insulin autoantibodies). All patients had a relative with type 2 diabetes. Two patients wear corrective lenses. Thyroid function tests were within normal ranges and anti-transglutaminase antibodies were absent in all patients. At baseline, all patients attended school without additional support. Urological consultations were routinely assessed during follow-up, with no evidence of neurogenic bladder in any patient. At the latest follow-up, all patients showed signs of puberty. During the initial follow-up, two patients experienced mild gastrointestinal adverse events: vomiting (*n* = 2), abdominal pain (*n* = 2), and nausea (*n* = 1). All symptoms resolved spontaneously. Serum biochemistry and hormonal laboratory tests were normal unless otherwise specified. Table 2 represents the patients' glucometabolic profiles.

**Table 1:** Patients' characteristics.

	**Patient 1**	**Patient 2**	**Patient 3**	**Patient 4**
Gender	Male	Female	Male	Male
Age at IDDM diagnosis, years	6.7	5.2	9	12.3
*WFS1* variant	c.409_424dup16; p.Val142fs*251 c.1628 T>G; p.Leu543Arg.	c.316-1G>A; c.757A>T p.Lys253Ter.	c.605A>G; p.Glu202Gly.c.1289C>T; p.Ser430Leu.	c.387G>A; p.Trp129. c.1675G>C; p.Ala559Pr.
Age at WS diagnosis, years	7.8	8.2	11.4	13.7
Age at baseline, years	11.3	10.7	12.3	14
Follow-up, months	27	20	16	8
Clinical features	IDDM, OA, Bilateral peripheral hearing loss, Diabetes insipidus, Hypergonadotropic hypogonadism	IDDM, OA	IDDM, OA	IDDM, OA

*IDDM, insulin-dependent diabetes mellitus; OA, optic nerve atrophy; WFS1, wolframin gene; WS, wolfram syndrome type 1*.

**Table 2:** Patients' glucometabolic profile.

	**Patient 1**				**Patient 2**			**Patient 3**			**Patient 4**	
Follow-up, sup^a^	0	7	16	27	0	8	20	0	9	16	0	8
Height, m	1.51	1.57	1.63	1.66	1.43	1.46	1.50	1.45	1.51	1.54	1.74	1.75
Weight, kg	49.3	53.1	57.4	54.1	29	29.1	30.6	56	59.4	68.8	56.3	55.1
BMI, kg/m^2^	21.7	21.5	21.6	19.7	14.2	13.7	13.6	26.5	26.2	29	18.8	18
sBMI^b^	0.82	0.61	0.48	−0.3	−2.07	−2.55	−2.87	1.59	1.47	1.92	−0.77	−1.17
HbA1c, %	8	7.9	8.5	7.5	6.7	6	6.6	7.5	8.1	9.4	8.5	7.1
HbA1c, mmol/mol	64	63	69	58	50	42	49	58	65	79	69	54
Insulin requirements, U/kg/day	1	1	1	0.47	0.28	0.35	0.38	0.5	0.5	0.78	0.4	0.08
Glucose TAR, %^c^	40	42	38	21	29	25	28	20	N/A^e^	70	26	38
Glucose TIR, %^d^	53	50	56	71	69	74	71	70	N/A^e^	29	73	62
Glucose TBR, %^f^	7	8	6	8	2	1	1	10	N/A^e^	1	1	0
Basal C-peptide, ng/mL	0.37	0.67	0.51	0.37	0.1	0.19	0.34	0.19	0.71	0.39	0.92	1.24
Basal glucose, mg/dL	170	142	150	101	87	95	124	157	177	151	180	150
Peak C-peptide, ng/mL	1.45	1.39	1.46	1.61	0.31	0.4	0.64	1.44	1.53	1.41	3.47	4.79
Peak Glucose, mg/dL	300	217	320	316	211	116	223	366	316	298	298	249
C-AUC, ng*min/mL	122	142	109	102	28	40	48	111	115	90	296	313

*BMI, body mass index; sBMI, standardized body mass index; HbA1c, glycated hemoglobin; TAR, time above range; TIR, time in range; TBR, time below range; C-AUC, C-peptide area under the curve*.

a *Follow-up at 0 months represents baseline*.

b *Regional growth charts are used for sBMI*.

c *Glucose time above range defined as glucose values >180 mg/dL*.

d *Glucose time in range defined as glucose values 70–180 mg/dL*.

e *At 9 months, patient 3 was non-compliant with glucometrics and thus data is not recorded*.

f *Glucose time below range defined as glucose values <70 mg/dL*.

### Case Descriptions

**Figure 1:**
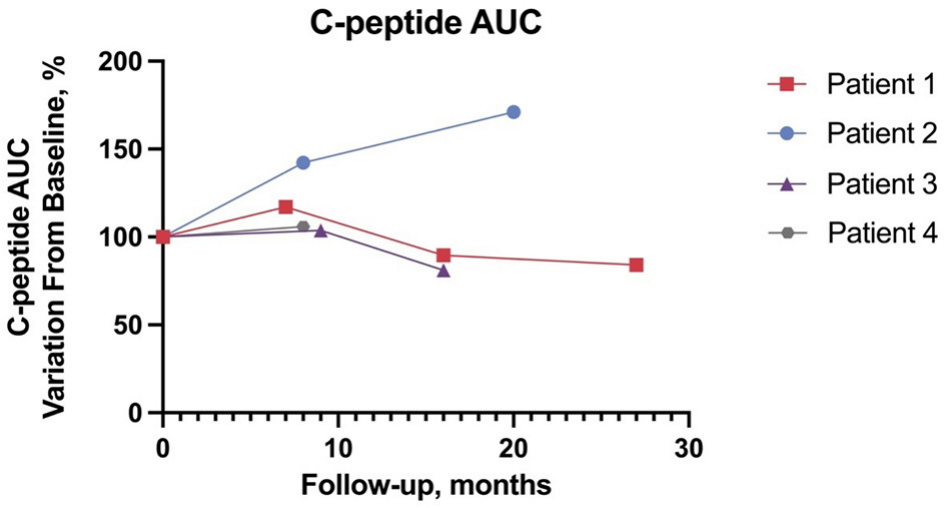
C-peptide area under the curve, percentage variation from baseline.

Patient 1 (male) presented with hyperopia and astigmatism at age 6. Shortly after, bilateral neurosensorial hearing loss was diagnosed and required the use of bilateral hearing aids. IDDM was diagnosed at age 6.7 years and treatment with multiple daily insulin injections (MDI) was initiated consisting of insulin aspart and glargine. Double heterozygous missense *WFS1* gene variants (c.409_424dup16; p.Val142fs^*^251 and c.1628 T>G; p.Leu543Arg) were confirmed at age 7.8 years with Sanger sequencing. One year later, patient 1 developed diabetes insipidus (see Table 1). At baseline, aged 11.3 years, with a standardized body mass index (sBMI) of 0.82, insulin requirements of 1 U/kg/day, patient 1 had a C-AUC of 122 ng^*^min/mL, HbA1c of 8% (64 mmol/mol), and a TIR of 53% and TAR of 40%. Liraglutide was initiated at 0.6 mg/day and the maximum dose of 1.8 mg/day was progressively achieved. As shown in Figure 1, at the latest 27-month follow-up C-AUC decreased to 102 ng^*^min/mL. Insulin requirements decreased to 0.47 U/kg/day, HbA1c improved to 7.5% (58 mmol/mol). Glucometrics via CGM showed improvements in TIR and TAR at 71% and 21%, respectively while TBR remained stable. The total daily insulin dose (TDD) was reduced by 53%. No adverse events occurred.

Patient 2 (female) had an unremarkable medical history except for transient mild speech delay (29). IDDM was diagnosed at age 5.2 years and insulin therapy was started with MDI consisting of insulin aspart and glargine. At age 8.2 years, two previously unreported likely pathogenetic variants (c.316-1G>A; c.757A>T and p.Lys253Ter), a splice site disruption and premature stop codon respectively, were identified by Next Generation Sequencing (NGS), and a WS diagnosis was confirmed. At baseline, aged 10.7 years, patient 2 presented with a sBMI of −2.08, insulin requirements of 0.28 U/kg/day, C-AUC of 28 ng^*^min/mL, HbA1c of 6.7% (50 mmol/mol), and a TIR of 69% recorded via CGM. Liraglutide was started at a dose of 0.3 mg/day and gradually increased to 1.8 mg/day. At 20 months, C-AUC increased to 48 ng^*^min/mL, TDD, TIR, TAR, TBR, and HbA1c remained stable. No severe adverse events were recorded.

Patient 3 (male) was diagnosed with IDDM at age 9 years and began insulin therapy with MDI consisting of insulin lispro and glargine. Prior to being diagnosed with IDDM, the patient had been complaining of visual impairment. Past medical history was unremarkable except for a transient mild motor delay (30). Family history was positive for bilateral congenital hypoacusia in his biological father and sister. WS diagnosis was established by NGS which confirmed a double heterozygous missense variant (c.605A>G; p.Glu202Gly and c.1289C>T; p.Ser430Leu) at age 11.4 years. At baseline, aged 12.3 years, patient 3 had a sBMI of 1.59, C-AUC of 111 ng^*^min/mL, HbA1c of 7.5% (58 mmol/mol) and insulin requirements of 0.5 U/kg/day. TIR, TAR, and TBR were 70, 20, and 10% respectively. Liraglutide was started at a dose of 0.3 mg/day and progressively increased to 1.8 mg/day as tolerated. At the 16-month follow-up the patient showed sBMI of 1.92, C-AUC of 90 ng^*^min/mL, HbA1c of 9.4% (79 mmol/mol), insulin requirements of 0.78 U/kg/day, and a TIR of 29%. TDD remained stable. No adverse events occurred.

Patient 4 (male) was diagnosed with IDDM at age 12.3 years and started insulin therapy with MDI consisting of insulin aspart and glargine. Prior to his diabetes diagnosis, the patient had an unremarkable past medical history. At age 13.4 years the patient complained of worsening vision and heterozygous WFS1 variants revealed a premature stop condon and missense variants (c.387G>A; p.Trp129 and c.1675G>C; p.Ala559Pr respectively) was confirmed with Sanger sequencing which revealed a double heterozygous variant. At baseline, age 14, patient 4 had a C-AUC of 296 ng^*^min/mL, HbA1c of 8.5% (69 mmol/mol), sBMI of −0.77 and insulin requirements of 0.4 U/kg/day. Glucometrics recorded TIR, TAR and TBR were 73%, 26%, and 1% respectively. Liraglutide was started at a dose of 0.6 mg/day and progressively increased to 1.8 mg/day as tolerated. At the latest 8-month follow-up, patient 4 had a sBMI of −1.17, C-AUC measuring 313 ng^*^min/mL, HbA1c improved to 7.1% (54 mmol/mol) and insulin requirements decreased to 0.08 U/kg/day. Glucometrics recorded TIR and TAR at 62% and 38%. TDD was reduced by 80%. No adverse events occurred.

### Neuro-Ophthalmology Results

**Figure 2:**
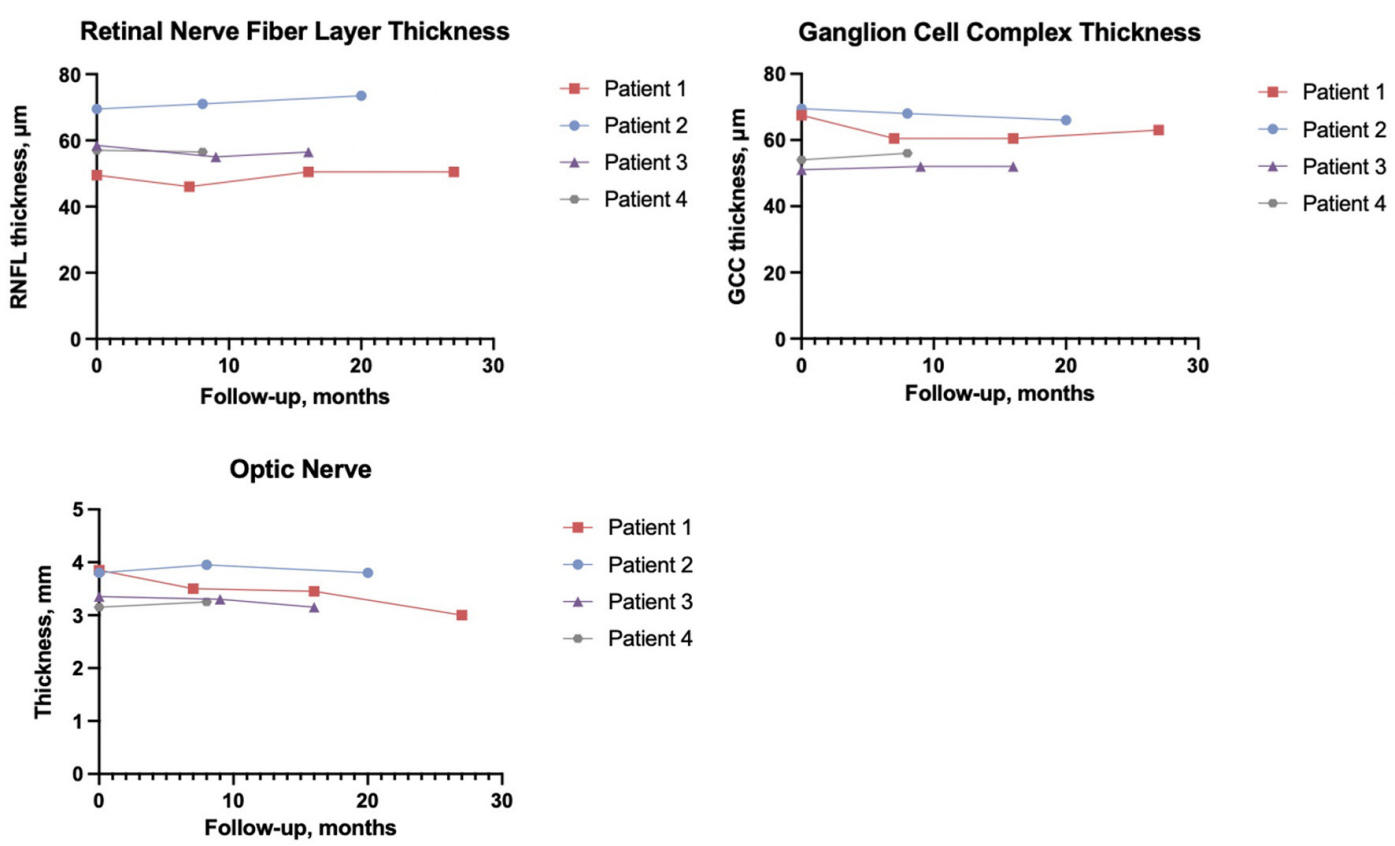
Average retinal nerve fiber layer thickness, ganglion cell complex thickness, and average optic nerve thickness are of both eyes.

Color blindness as measured by Ishihara's test, showed color blindness was present at baseline in two male patients (1 and 3) while patient 4 worsened from baseline, patient 2 remained normal. Visual field tests revealed different patterns of defects, which included generalized loss of the visual field, inferior and superior arcuate scotomas, and ring scotomas. PERG N95/P50 values were found to have increased during follow-up in 5/6 eyes (unavailable baseline values in 2 eyes) (31). The RNFL and GCC (Figures 2A,B) were recorded below reference ranges and remained stable during the follow-up. In all patients, intraocular pressure was normal, and no signs of ocular anterior segment alterations were seen neither at baseline visit nor at the latest follow-up visits. See electronic supplementary files for extended neuro-ophthalmology methods (Supplementary Methods; Supplementary Table S1).

### Neuroradiology Results

Previous studies have shown that classical neuro-radiological manifestations of WS include atrophy of the brainstem, diffuse cerebellar gray, and white matter atrophy, thinning of the middle cerebellar peduncle, absent posterior pituitary, and optic nerve and optic tract atrophy (32, 33). At baseline, we observed that all patients showed signs of atrophy of the intracranial and intraorbital optic nerves; patient 1 and patient 3 also showed atrophy of the optic chiasm. Mild brainstem/pontine atrophy was evident in all patients; patient 1 also displayed atrophy of the superior cerebellar peduncle and the cerebellum, along with the absence of spontaneous hyperintensity of the pituitary gland posterior lobe. Figure 2C represents the trend of the optic nerves thickness. Of note, in all patients, the neuroradiological picture remained unchanged at the latest follow-up. See electronic supplementary files for the extended neuroradiology findings (Supplementary Table S2; Supplementary Figure S1).

### Neurophysiology Results

The mfVEPs are shown in the electronic supplementary files labeled Supplementary Figure S2. A heterogeneous trend was observed among the four patients, as patient 1 remained stable, patient 2 and 4 showed transiently increased amplitude, and Patient 3 worsened. Of note, the transient amplitude increase observed in patient 2 was associated with a transitory improvement in LVCA, as shown in Supplementary Figure S3 in the electronic supplementary files.

## Discussion

We present preliminary data on the safety, tolerability, and efficacy of long-term off-label liraglutide treatment involving four pediatric patients affected by WS. All patients had IDDM and optic atrophy; patients 1, 3, and 4 were carriers of *WFS1* pathogenic variants, while the variants found in patient 2 were not previously described and classified as presumably pathogenetic due to splice acceptor site disruption (c.316-1G>A) and insertion of a premature stop codon (c.757A>T) (8). Liraglutide was well-tolerated by all patients up to the maximum dosage of 1.8 mg/day, and at the latest 8–27-month follow-up. Gastrointestinal adverse events were mild and transient. We found no alteration in pancreatic enzymes and thyroid hormones, nor other abnormal findings (34). Liraglutide has been associated with weight loss, which is a potential concern in administration to non-obese patients (35). While sBMI of patients 1, 2, and 4 decreased, it increased in patient 3 along with worsening of glucometrics, underlying the importance of a concomitant healthy diet and lifestyle. At the latest time point, C-AUC ranged between 81% and 171% of baseline, suggesting no evident beta cell deterioration during treatment. TDD showed a significant reduction in 2 patients. TBR improved in one patient, while changes in TIR were heterogeneous. Importantly, no significant changes in ophthalmological, neuroradiological, neurophysiological parameters were observed during follow-up and no new Wolfram-associated features were diagnosed during the follow-up. WS is a life-limiting and lethal disease with no cure. GLP-1RAs are known to promote beta cell glucose metabolism and survival (22, 36). We provide the first preliminary report on the use of liraglutide in pediatric WS patients. In our cohort liraglutide was safe and well-tolerated. We observed no deterioration in C-peptide secretion and OCT parameters, which are important markers of disease progression, while not observing the onset of new WS-related symptoms. Of note, also Valproate (NCT03717909) and Dantrolene (NCT02829268) are under investigation as possible therapeutic options for WS (37–40). WS is a rare orphan disease; consequently, this study suffers from the intrinsic limitations of the absence of a control group, a small sample size. The current absence of a clear genotype-to-phenotype correlation benchmark in terms of clinical progression is another significant limitation to the interpretation of our results. Furthermore, our follow-up may still be relatively too short to demonstrate significant changes. We hypothesize that adjunctive treatment with liraglutide may also have indirect beneficial effects in WS progression due to improved glucometrics, which in turn may reduce diabetes-related oxidative stress (41). Within our small case series, it is difficult to conclude whether adjunctive treatment with liraglutide may have had a significant impact on glucose metabolism as outcomes varied between patients. However, these variations may also depend on other factors such as lifestyle habits and adherence to daily diabetes management. Of note, patient 3 had the worst BMI, did not adhere to CGM, and concomitantly showed the poorest glucose control. On the other hand, all CGM-wearing patients showed improved glucometrics from baseline. In conclusions, the rational repurposing of liraglutide is supported by encouraging preliminary safety and efficacy data, thus providing the basis for further confirmatory studies; comparison with a cohort of natural history patients may prove useful as a control for future studies.

## Data Availability Statement

The original contributions presented in the study are included in the article/Supplementary Material, further inquiries can be directed to the corresponding author.

## Ethics Statement

The studies involving human participants were reviewed and approved by Comitato Etico per l'assistenza e per la ricerca, IRCCS Ospedale San Raffaele. Written informed consent to participate in this study was provided by the participants' legal guardian/next of kin.

## Author Contributions

All authors listed have made a substantial, direct, and intellectual contribution to the work and approved it for publication.

## Conflict of Interest

The authors declare that the research was conducted in the absence of any commercial or financial relationships that could be construed as a potential conflict of interest.

## Publisher's Note

All claims expressed in this article are solely those of the authors and do not necessarily represent those of their affiliated organizations, or those of the publisher, the editors and the reviewers. Any product that may be evaluated in this article, or claim that may be made by its manufacturer, is not guaranteed or endorsed by the publisher.
