# Supplementary material for: Case Report: Off-Label Liraglutide Use in Children With Wolfram Syndrome Type 1: Extensive Characterization of Four Patients
Source: Front Pediatr. 2021 Dec 14;9:755365. doi: 10.3389/fped.2021.755365 (PMC8712700; doi:10.3389/fped.2021.755365)
Supplement: Supplementary file 1 [file Data_Sheet_1.docx]

**Supplementary materials**

**eMethods:**

**Extended Neuro-ophthalmology methods:**

Best-Corrected Visual acuity measurement (BCVA)

The BCVA was assessed by the modified Early Treatment Diabetic Retinopathy Study table (Lighthouse, Low Vision Products, Long Island City, NY) at a distance of 4 m. The visual acuity was measured as logMAR values.

Pattern Electroretinography (PERG)

The PERG was obtained through a bipolar method between the stimulated and the patched eye. The acquired signal was then amplified and averaged reducing artifacts through BM600 (Biomedica Mangoni, Pisa, Italy). The analysis time was 250 msec. Consequent waves with peaks of negative, positive, and negative polarity represent a normal transient PERG response. In our analysis of PERG data, the peak-to-peak amplitude between the P50 and the N95 peaks was considered: PERG N95-P50 amplitude measured in microvolts.

Ishihara test, Humphrey 30-2 visual field examination (Humphrey Field Analyzer [HFA] 740; Zeiss, San Leandro, CA), slitlamp biomicroscopy, intraocular pressure measurement, and indirect ophthalmoscopy following were conducted according to standard local procedure. eTable 1 represents extended neuro-ophthalmological results.

|  | | **Follow-up, months**^a^ | **BCVA, LogMAR** | **Ishihara Test** | **MD value, dB** | **Average RNFL thickness, μm** | **Average GCC thickness, μm** | **PERG (N95/P50), μV** |
| --- | --- | --- | --- | --- | --- | --- | --- | --- |
| **Patient 1** | Right eye | 0^a^ | 0.7 | 0/14 | -24.91 | 53 | 64 | 0.07 |
|  |  | 7 | 0.7 | 0/12 | -27.89 | 47 | 53 | 0.12 |
|  |  | 16 | 0.7 | 0/12 | -18.28 | 51 | 66 | 0.5 |
|  |  | 27 | 0.7 | 0/12 | -30.41 | 49 | 61 | 0.21 |
|  | Left Eye | 0^a^ | 0.7 | 1/14 | -28.57 | 46 | 71 | 0.19 |
|  |  | 7 | 0.7 | 0/12 | -24.69 | 45 | 68 | 0.35 |
|  |  | 16 | 0.6 | 0/12 | -14.37 | 50 | 55 | 0.32 |
|  |  | 27 | 0.6 | 0/12 | -25.72 | 52 | 65 | 0.34 |
| **Patient 2** | Right eye | 0^a^ | 0 | 14/14 | -6.09 | 71 | 70 | 0.26 |
|  |  | 8 | 0 | 12/12 | -6.94 | 74 | 67 | 0.29 |
|  |  | 20 | 0 | 12/12 | -6.44 | 73 | 66 | 0.3 |
|  | Left eye | 0^a^ | 0 | 14/14 | -6.56 | 68 | 69 | 0.38 |
|  |  | 8 | 0 | 12/12 | -5.82 | 68 | 69 | 0.47 |
|  |  | 20 | 0 | 12/12 | -6.81 | 74 | 66 | 0.65 |
| **Patient 3** | Right eye | 0^a^ | 0.7 | 0/12 | -5.75 | 58 | 52 | 0.82 |
|  |  | 9 | 0.7 | 0/12 | -5.96 | 54 | 53 | N/A^b^ |
|  |  | 16 | 0.5 | 0/12 | -6.47 | 56 | 52 | 0.02 |
|  | Left eye | 0^a^ | 0.7 | 0/12 | -7.36 | 59 | 50 | 0.31 |
|  |  | 9 | 0.7 | 0/12 | -6.14 | 56 | 51 | N/A^b^ |
|  |  | 16 | 0.7 | 0/12 | -6.9 | 57 | 52 | 0.02 |
| **Patient 4** | Right eye | 0^a^ | 0.1 | 8/12 | -3.23 | 58 | 55 | N/A^b^ |
|  |  | 8 | 0.1 | 5/12 | -5.02 | 56 | 56 | 0.4 |
|  | Left eye | 0^a^ | 0.1 | 7/12 | -3.05 | 56 | 53 | N/A^b^ |
|  |  | 8 | 0.1 | 4/12 | -5.23 | 57 | 56 | 0.36 |
| Abbreviations: BCVA, best corrected visual acuity; MD, mean deviation: RNFL, retinal nerve fiber layer thickness; GCC, ganglion cell complex; PERG, pattern electroretinography.  ^a^ Follow-up at 0 months represents baseline.  ^b^ Patient was not able to conduct the PERG due to technical difficulties. | | | | | | | | |

eTable 1 Neuro-ophthalmological results

| T | **Patient 1** | | | | **Patient 2** | | | **Patient 3** | | | **Patient 4** | |
| --- | --- | --- | --- | --- | --- | --- | --- | --- | --- | --- | --- | --- |
| Follow-up, months^a^ | 0 | 7 | 16 | 27 | 0 | 8 | 20 | 0 | 9 | 16 | 0 | 9 |
| Right optic nerve, mm | 4 | 3.2 | 3.1 | 3 | 3.8 | 3.8 | 3.8 | 3.5 | 3.4 | 3.3 | 3 | 3.1 |
| Left optic nerve, mm | 3.7 | 3.8 | 3.8 | 3 | 3.8 | 4.1 | 3.8 | 3.2 | 3.2 | 3 | 3.3 | 3.4 |
| Right optic tract, mm | 2.1 | 2.5 | 2.3 | 2 | 3 | 3.6 | 2.8 | 2.6 | 2.5 | 2.3 | 2.5 | 2.5 |
| Left optic tract, mm | 1.9 | 2.5 | 2.4 | 2.3 | 3.3 | 3.3 | 2.9 | 2.2 | 2.4 | 2.4 | 2.9 | 2.9 |
| Chiasmatic right-left dimension | 10.6 | 11 | 11 | 10.8 | 11.3 | 10.5 | 10.1 | 10.1 | 10.3 | 10.3 | 12.2 | 12.7 |
| Central chiasmatic height | 2.6 | 2.2 | 1.8 | 1.7 | 2.8 | 2.6 | 2.6 | 2.5 | 2.2 | 1.8 | 1.5 | 1.6 |
| Right chiasmatic height | 2.5 | 2.4 | 2 | 1.9 | 2.7 | 2.5 | 2.6 | 2.5 | 2.2 | 1.8 | 1.6 | 1.5 |
| Left chiasmatic height | 3.3 | 2.2 | 1.7 | 1.7 | 2.9 | 2.6 | 2.1 | 2.3 | 2.2 | 1.9 | 1.5 | 1.3 |
| Periventricular T2 hyperintensity^b^ | 0.5 | 0.5 | 0.5 | 0.5 | 0.5 | 0.5 | 0.5 | 0.5 | 0.5 | 0.5 | 0 | 0 |
| Pons T2 hyperintensity^b^ | 0.5 | 0.5 | 0.5 | 0.5 | 0 | 0.5 | 0.5 | 0 | 0 | 0 | 0 | 0 |
| ^a^ Follow-up at 0 months represents baseline.  ^b^ Extent of signal alteration of the periventricular and pontine white matter 0=absent, 1=present, and 0.5=doubtful. | | | | | | | | | | | | |

eTable 2. Magnetic Resonance Imaging measurements


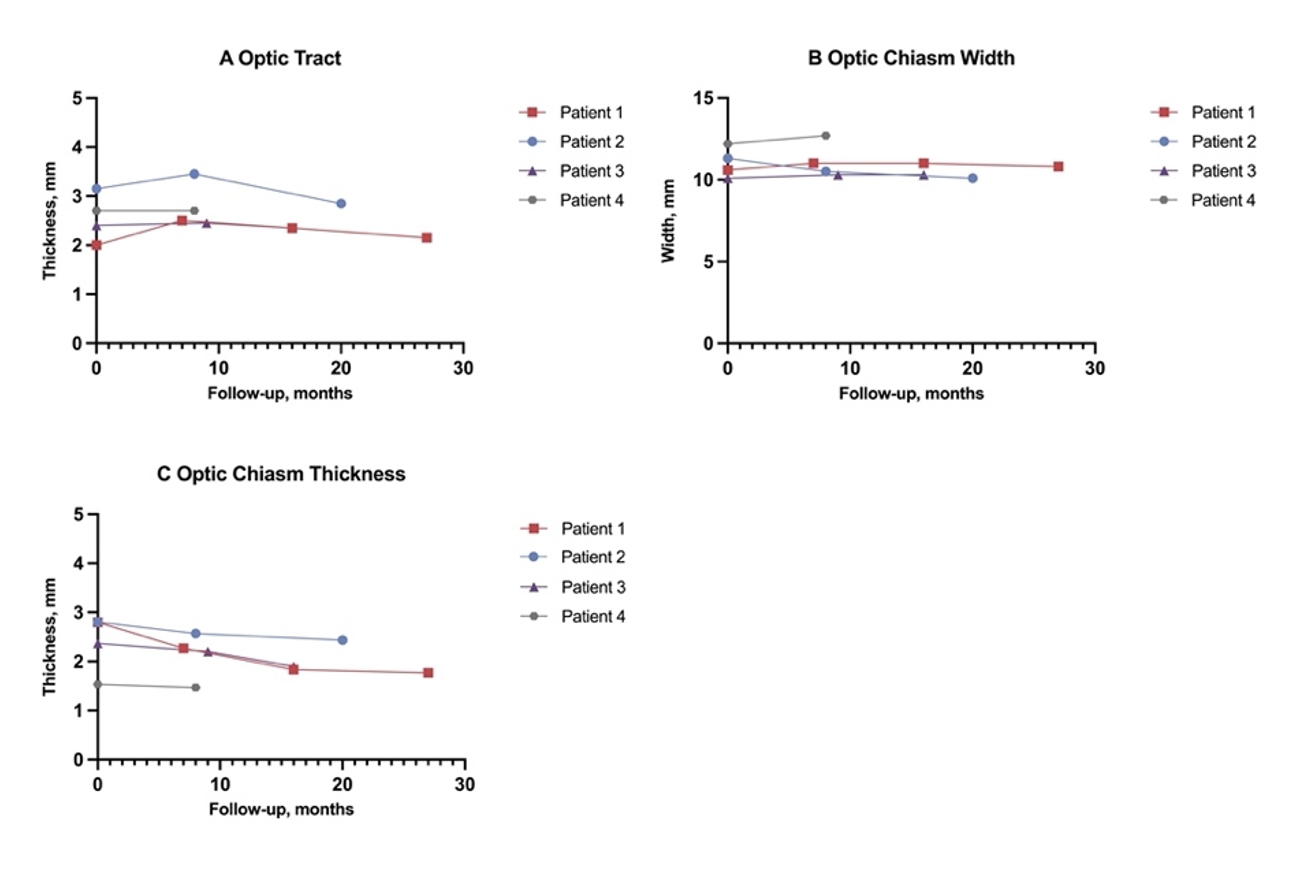


eFigure 1. Neuroradiology findings: Average of Optic tract (A), Optic Chiasm width (B), and Optic Chiasm thickness (C).


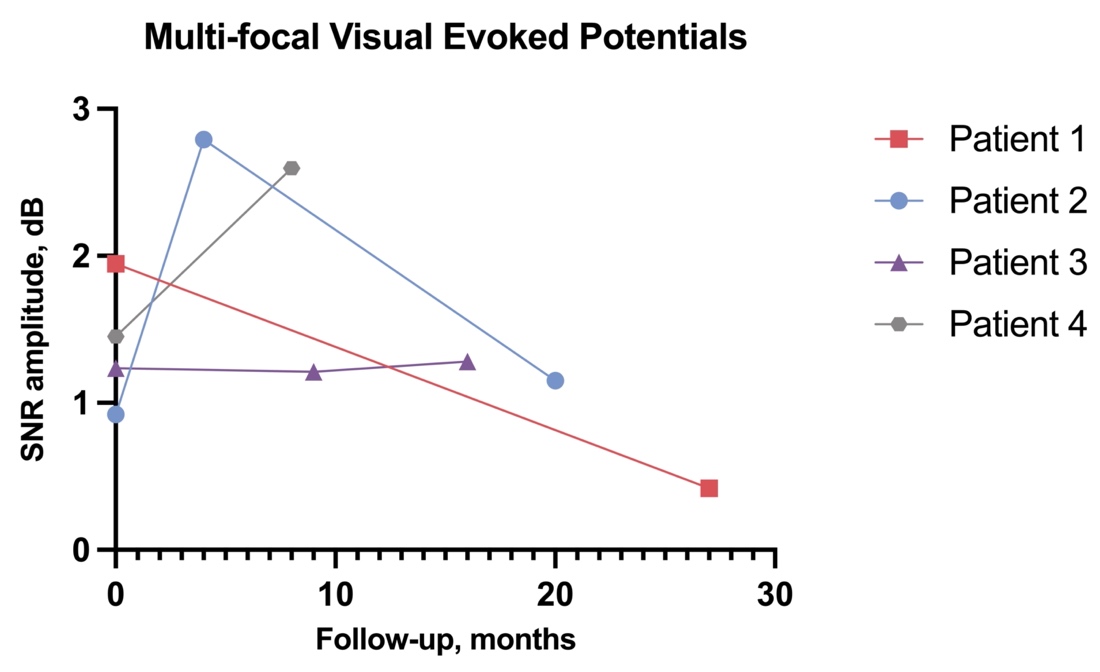


eFigure 2. Multi-focal Visual Evoked Potentials

eFigure 3. Low Contrast Visual Acuity
